# Stress and the CRH System, Norepinephrine, Depression, and Type 2 Diabetes

**DOI:** 10.3390/biomedicines12061187

**Published:** 2024-05-27

**Authors:** Michele Perrelli, Pruthvi Goparaju, Teodor T. Postolache, Laura del Bosque-Plata, Claudia Gragnoli

**Affiliations:** 1 ASL Roma 1, Santo Spirito Hospital, 00193 Rome, Italy; micheleperrelli@hotmail.com; 2 Division of Endocrinology, Department of Medicine, Creighton University School of Medicine, Omaha, NE 68124, USA; pruthvigoparaju@creighton.edu; 3 Mood and Anxiety Program, Department of Psychiatry, University of Maryland School of Medicine, Baltimore, MD 21201, USA; tpostola@som.umaryland.edu; 4 Rocky Mountain Mental Illness Research Education and Clinical Center (MIRECC), Veterans Integrated Service Network (VISN) 19, Military and Veteran Microbiome: Consortium for Research and Education (MVM-CoRE), Aurora, CO 80246, USA; 5 Mental Illness Research Education and Clinical Center (MIRECC), Veterans Integrated Service Network (VISN) 5, VA Capitol Health Care Network, Baltimore, MD 21090, USA; 6 Nutrigenetics, and Nutrigenomic Laboratory, National Institute of Genomic Medicine, Mexico City 14610, Mexico; ldelbosque@inmegen.gob.mx; 7 Department of Public Health Sciences, Penn State College of Medicine, Hershey, PA 17033, USA; 8 Klinik für Endokrinologie, Diabetologie und Klinische Ernährung, Universitätsspital Zürich, 8091 Zürich, Switzerland; 9 Molecular Biology Laboratory, Bios Biotech Multi-Diagnostic Health Center, 00197 Rome, Italy

**Keywords:** CRH, corticotropin releasing hormone receptor, norepinephrine, autonomous sympathetic nervous system, major depressive disorder, depression, type 2 diabetes, HPA axis, stress, cortisol

## Abstract

Major depressive disorder (MDD) increases the risk of type 2 diabetes (T2D) by 60% in untreated patients, and hypercortisolism is common in MDD as well as in some patients with T2D. Patients with MDD, despite hypercortisolism, show inappropriately normal levels of corticotropin-releasing hormone (CRH) and plasma adrenocorticotropin (ACTH) in the cerebrospinal fluid, which might implicate impaired negative feedback. Also, a positive feedback loop of the CRH–norepinephrine (NE)–CRH system may be involved in the hypercortisolism of MDD and T2D. Dysfunctional CRH receptor 1 (CRHR1) and CRH receptor 2 (CRHR2), both of which are involved in glucose regulation, may explain hypercortisolism in MDD and T2D, at least in a subgroup of patients. CRHR1 increases glucose-stimulated insulin secretion. Dysfunctional *CRHR1* variants can cause hypercortisolism, leading to serotonin dysfunction and depression, which can contribute to hyperglycemia, insulin resistance, and increased visceral fat, all of which are characteristics of T2D. CRHR2 is implicated in glucose homeostasis through the regulation of insulin secretion and gastrointestinal functions, and it stimulates insulin sensitivity at the muscular level. A few studies show a correlation of the *CRHR2* gene with depressive disorders. Based on our own research, we have found a linkage and association (i.e., linkage disequilibrium [LD]) of the genes *CRHR1* and *CRHR2* with MDD and T2D in families with T2D. The correlation of *CRHR1* and *CRHR2* with MDD appears stronger than that with T2D, and per our hypothesis, MDD may precede the onset of T2D. According to the findings of our analysis, *CRHR1* and *CRHR2* variants could modify the response to prolonged chronic stress and contribute to high levels of cortisol, increasing the risk of developing MDD, T2D, and the comorbidity MDD-T2D. We report here the potential links of the CRH system, NE, and their roles in MDD and T2D.

## 1. Introduction

### 1.1. Major Depressive Disorder (MDD) and Type 2 Diabetes (T2D) Prevalence

Both MDD and T2D are widespread global pathologies. Worldwide, about 5% of adults suffer from depression [1], and by 2030, depression is estimated to become the most common disease in the world, according to the World Health Organization [2]. Meanwhile, worldwide, about 10% of adults suffer from T2D [3], with 58% of European Caucasians having impaired fasting glucose (glycemia ≥ 100 mg/dL) [4]. This perspective article explores what is known about the role of linkage and association (i.e., linkage disequilibrium [LD]) of the corticotropin-releasing hormone (CRH) *CRHR1* and *CRHR2* genes with MDD and T2D in families with T2D. We searched within PubMed using the keywords “type 2 diabetes, T2D, major depressive disorder, MDD, depression, stress, hypothalamic-pituitary-adrenal axis, HPA, CRH, CRHR, norepinephrine” and included articles reporting valuable concepts on the association of chronic stress, the CRH system, norepinephrine (NE), depression, and T2D, including the genes *CRHR1* and *CRHR2*.

### 1.2. MDD and T2D Comorbidity

MDD and T2D are often associated, and numerous different genes may underlie their comorbid pathogenesis. As MDD increases the risk of T2D by 60% [5] in patients never previously treated pharmacologically for MDD, genetic predisposition may—at least partially—underlie the MDD-T2D comorbidity, not depending on antidepressant therapy [6]. Conversely, T2D increases the risk for MDD, albeit slightly [5]. Furthermore, depressive symptoms increase both the risk for T2D and, in diabetic patients, the increase in blood sugar levels and the risk of complications and mortality from all causes [7]. While many genes and neuro-endocrine-metabolic pathways are likely implicated in T2D, MDD, and their comorbidity, the hypothalamic–pituitary–adrenal (HPA) axis and the serotonergic system are known to be interconnectedly involved in MDD [8,9,10,11,12,13,14,15] and perhaps also in T2D and associated metabolic alterations [11,15,16,17,18,19]. In fact, the etiology of stress in the pathogenesis of T2D has been reported [20,21]. Also, subjects with depression have higher hair cortisol concentrations than healthy subjects [22]. Similarly, in a cohort of African American adults with and without T2D, elevated hair cortisol, a marker of long-term HPA axis dysregulation, was correlated with increased glycosylated hemoglobin (HbA1c) in the whole group and in the T2D group [19].

Chronic stress acts on the gut–brain axis and has systemic effects, such as the induction of systemic inflammation present in both depression and T2D. Thus, another potential mechanism is represented by the stress-related intestinal microbiota imbalance—also named dysbiosis—which, by impairing the gut–brain–immune axis, can lead to stress-related MDD-T2D comorbidity [23]. In addition, the persistent activation of the sympathetic nervous system (SNS) results in increased levels of proinflammatory cytokines, which *per se* can trigger the HPA axis hyper-response during stress. Furthermore, in the presence of hyperglycemia and/or T2D, the immune system can be dysregulated, thereby augmenting cytokine release, which can increasingly stimulate the HPA axis and can contribute to depression, T2D, and their comorbidity [24].

Furthermore, genetic studies on MDD-T2D comorbidity will disclose mechanisms underlying T2D and MDD and their comorbidity. Finally, confirmation in larger ethnically diverse samples and longitudinal studies will lead to primary prevention targeting individuals at risk for both T2D and MDD and secondary prevention for patients with a confirmed diagnosis of T2D–MDD.

### 1.3. Hypothalamic–Pituitary–Adrenal (HPA) Axis and Stress

The HPA axis is a neuroendocrine system that regulates the stress response and interacts with the serotonergic, noradrenergic, and dopaminergic brain systems.

In response to prolonged chronic stress, the hypothalamic paraventricular nucleus (PVN) secretes corticotropin-releasing hormone (CRH, aka corticotropin-releasing factor, CRF), which in turn stimulates pituitary corticotropin (ACTH) secretion, resulting in an increased in plasma cortisol produced by the adrenal gland [25]. The PVN also receives circadian signals from the hypothalamic suprachiasmatic nucleus connected to the retina through the retinohypothalamic tract, which also functions as the central circadian clock [26].

Corticotropinergic neurons are present not only in the anterior pituitary gland but also in the hypothalamus, hippocampus, amygdala, and locus coeruleus (LC), located on the floor of the fourth ventricle. CRHR1 and CRHR2 are G-protein-coupled receptors carrying an amino acid sequence homology of 71% [27], binding CRH and stimulating ACTH secretion. CRHR1 and CRHR2 are responsible for the HPA axis response to stress and circadian rhythms. CRHR1 mostly binds CRH, while CRHR2 binds urocortin with an affinity 40 times greater than CRH [28]. *CRHR1* is expressed especially in the hippocampus and generally in the brain, and to a lesser extent in peripheral tissues, such as the adipose tissue and liver. *CRHR2* is mostly expressed in peripheral tissues such as the liver and adipose tissue but is also expressed in the brain [28].

### 1.4. Resilience, Stress, CRH System, and Depression

Resilience varies among human and non-human primates. Anxious temperament (AT) manifests early in life with physiological and behavioral hyper-reactivity to mildly threatening stimuli. In children, AT predicts psychopathology and risk for anxiety disorders and depression. In monkeys, the function of the anterior hippocampus and the central nucleus amygdala predicts AT, and even if anatomically closely linked, their heritability differs significantly, as the anterior hippocampus metabolic activity is significantly more heritable compared to the central nucleus amygdala metabolic activity, indicating dissimilar influences of genes and environment mediating AT and anxiety and depression risk [29].

Various studies show that the genetic basis of depression may reside, in part, in genes regulating stress-response systems [12,30] such as the HPA axis [31,32]. Various endocrine factors, including CRH and glucocorticoids, have been implicated in the structural and intracellular abnormalities seen in depression [33].

Early life adversity (e.g., early life maternal separation) [34] increases HPA axis activity in rats and humans [31,34] and generates anxious and depressive behaviors in adult mice [35].

In humans, childhood events, like abuse or loss, are closely related to the risk of adult MDD [36]. Early life stress, characterized by altered neural plasticity, leads in subjects with reduced resilience and additional stressors to persistent central nervous system (CNS)–CRH circuit hyperactivity, increased HPA axis sensitization, and sympathetic nervous system (SNS) response. This lasting increased stress sensitization mediates vulnerability to adolescent and adult depression [37]. Furthermore, the CRH system plays a role in psychopathology, anxiety, and depressive disorders triggered by prolonged chronic stress, and various neuro-behavioral-endocrine-sympathetic-immune responses intensely involve the CRH system [31].

### 1.5. CRH System, Stress, and CRH–Norepinephrine–CRH Circuit

Cortisol secretion accompanying acute stress is a physiological coping response to anxiety triggers. Catecholamine secretion, specifically the major SNS neurotransmitter, norepinephrine (NE), regulated by the catechol-o-methyltransferase (COMT), mediates SNS activation during short-term stress [38]. During induced stress, human bilateral amygdala activity strongly mediates a sympathetic response and attachment insecurity. Thus, under stress, the amygdala activates the central SNS [39]. The brainstem SNS lies in the LC, the main site synthesizing NE, beyond the adrenal medulla. Also, cortical and midbrain areas have noradrenergic neuron projections. To maintain homeostasis, chronic stress usually elicits central nervous system adaptation. Extrahypothalamic CRH and LC-NE systems are the principal components of stress response. In animals, CRH produces various anxiety-, arousal-, and stress-associated behaviors. In the LC, CRH increases the activity of tyrosine hydroxylase (rate-limiting enzyme in the synthesis of catecholamines) and therefore increases the release of NE in the projection areas of the LC. Chronic “unpredictable” stress can maintain CRH neuronal activity and LC-NE system dysregulation, impairing the adaptation system. Further, the NE-CRH interaction may occur in various brain areas, including the hypothalamic PVN and the amygdala central nucleus, where NE stimulates CRH release. The CRH–NE–CRH feed-forward system progressively augments the stress response with repeated exposure. Acute and chronic moderate and “predictable” stresses induce beta-adrenergic receptor down-regulation, which represents stress adaptation. On the contrary, chronic “unpredictable” stress, resembling a model for depression, up-regulates the beta-adrenergic receptor [40]. In rats exposed to 14-day-long chronic “unpredictable” stress, treatment with the selective serotonin-reuptake inhibitor (SSRI) citalopram causes frontal cortex beta-receptor down-regulation; thus, increased 5-hydroxytryptophan (5-HT) availability can preserve beta-receptor down-regulation by NE-potentiating agents. In human depression, CRH–NE hyperactivation of the CRH–NE feed-forward system is implicated in sympathetic activation, hyperarousal, and anxiety. 5-HT reduction is implicated in depression, and an SSRI-mediated 5-HT increase may normalize beta-receptor down-regulation, offering, in prolonged stress, adaptive self-regulation against excessive CRH–NE activity [40]. In cattle, intravenous tryptophan administration attenuates cortisol secretion induced by the intracerebroventricular injection of NE [41].

## 2. Stress, Depressive Symptoms, T2D, and Our Hypothesis

We hypothesize that a group of depressed patients may have an underlying genetic predisposition to augmented, maladaptive hyperarousal and stress vulnerability activating and self-feeding the CRH–NE–CRH system, characterized by defective CRHR1 and high-normal CRH and NE levels, the latter triggering peripheral cortisol secretion, in the absence of a clear ACTH augmentation. Another theory, depending on the depressed patient subgroups, is that long-lasting chronic stress may be maintained by an enduring imbalance of the HPA axis via CRH and ACTH secretion and hypercortisolism. As the latter can also be triggered by central NE and peripheral adrenal catecholamine secretion, the two hypothesized mechanisms may overlap in some patients presenting with increased ACTH levels beyond increased CRH, NE, and cortisol levels.

**Figure 1:**
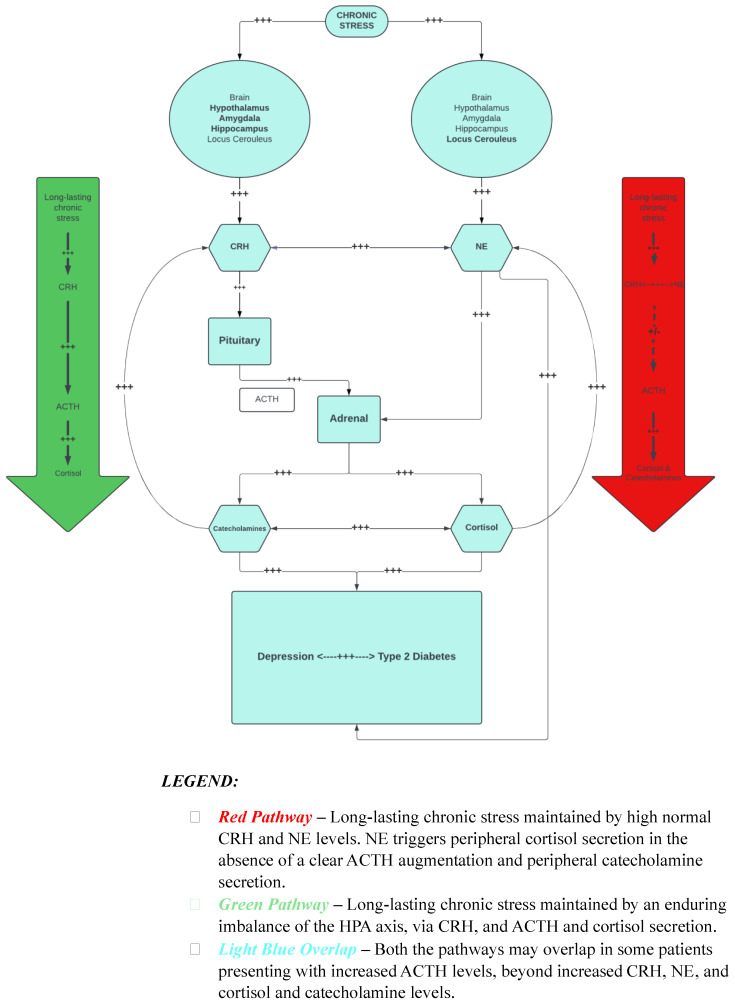
CRH-NE-CRH Circuit and Hypothalamic-Pituitary-Adrenal Axis Implication in the Comorbidity of Depression and Type 2 Diabetes.

Figure 1 illustrates these concepts for how stress and the HPA axis relate to T2D and depression. We hypothesize that receptor resistance of the CRH system can induce hypercortisolism: the CRH system is implicated in the response to depression and prolonged stress [30,32], and *CRH* SNPs are variants of depression risk [32]. Depressed and hypercortisolemic subjects have an attenuated ACTH response to CRH, thus potentially suggesting intact negative feedback. CRH infusion in healthy individuals induces hypercortisolism as in depression but via ACTH [42]. This difference suggests that in depression, hypercortisolism may be due to a defect in the CRHR receptor response with the hypersecretion of CRH and consequently of NE.

A significant cortisol response to a blunted ACTH response may also indicate that the adrenal glands hyper-respond to ACTH due to an ACTH receptor or melanocortin receptor 2 (MC2R) abnormality [43]. The most probable dynamics, however, would be the increase in central NE secretion, triggered by the limbic response to CRH [44]; in turn, NE stimulates the secretion of adrenal cortisol [41,45] and, through positive feedback, also the hypothalamic and limbic secretion of CRH, supporting the central hyperactivation of the HPA system [46,47]. This hypothetical mechanism fully fits with the strong correlation of plasma cortisol with cerebrospinal fluid (CSF) NE in depressed patients [48].

In depressed and control subjects, circadian variations of CSF NE and plasma cortisolemia are superimposable and correlate positively during the day. While healthy individuals have significantly negatively correlated plasma cortisol and CSF-CRH levels, depressed patients have significantly higher circadian CSF NE and plasma cortisol levels, but inappropriately “normal” plasma ACTH and CRH [44]. These studies compellingly suggest that upstream CRH receptor dysfunction may be a cause of depression and that downstream hypercortisolism may further feed into depression by altering the serotonin pathway [14], as well as increasing the risk of T2D and accounting, at least in part, for MDD-T2D comorbidity. The above-mentioned robust correlation of cortisolemia with CSF NE of depressed patients [48] points towards a persistent stress-response dysregulation in depression, independently from any conscious stress, and indicates a bidirectional mutual boosting link between the hyper-noradrenergic state and the hyperfunctioning HPA axis, each triggered and maintained by hypercortisolism through interactions at the central level and the peripheral adrenal level [48].

Increased cortisol and reduced HPA axis feedback are alterations present in both depression and T2D [49,50], while T2D, metabolic traits, and MDD are associated with hypercortisolemia [11,15]. The increase in cortisol due to chronic stress, according to our hypothesis, could also lead to T2D. However, anomalies in the feedback and activity of the HPA axis are characteristics found in aging [18].

Also, a potential mechanism may be related to maternal nutritional imbalances, which may permanently affect the offspring by altering the cortisol and sympathetic stress response. Recent studies showed that lower human birth weight—a marker of fetal undernutrition—is correlated in both children and adults to impaired sympathetic nervous system and HPA axis stress responses, which are further linked to depression and T2D [51].

A genetic predisposition to HPA axis hyperactivation and hypercortisolemia could induce glucose intolerance [52], metabolic abnormalities, and depressive symptoms [11]. Various HPA axis receptor genes are related to metabolic alterations [43,53]. Furthermore, hypercortisolemia causes an alteration in serotonergic transmission, which is one of the determinants of depression [12].

To support our hypothesis, studies have shown that CRH injected in rats’ brains increases plasmatic epinephrine, NE, and glucagon, leading to hyperglycemia. Of interest, CRH-induced hyperglycemia is present even with hypophysectomy or adrenalectomy; thus, this disease model is not due to the HPA axis but to CRH enhancing both epinephrine and NE secretion [44]. Of note, in congenital adrenal hyperplasia due to 21-hydroxylase deficiency, cortisol precursors are increasingly secreted with ACTH stimulation, indicating impaired cortisol production and compensating increased hypothalamic CRH secretion. In a study, carriers of 21-hydroxylase deficiency (i.e., parents of children with classical congenital adrenal hyperplasia) showed significantly higher state-anxiety scores than healthy subjects, and their mean 24 h urinary free cortisol excretion was significantly associated with psychoticism and paranoid ideation, thereby making them susceptible to anxiety disorders [54].

Of note, the prevalence of subclinical hypercortisolism was found to be significantly higher in hospitalized patients with T2D compared to controls, with 7% of T2D statistically attributable to subclinical hypercortisolism, and subclinical hypercortisolism was significantly related to severe T2D, defined by the presence of insulin treatment, hypertension, and dyslipidemia [55].

A study reported that 30% of T2D patients have significantly elevated basal plasma cortisol levels, higher cortisol levels after a dexamethasone (DEX) suppression test, and a larger response to CRH, without significantly higher ACTH levels. The increased responsiveness to CRH, together with reduced suppression after DEX, may indicate dysfunctional negative HPA axis feedback in T2D. An exaggerated HPA response to the CRH test is also present among depressed patients and the elderly [56,57] and could imply impaired feedback due to a hippocampal glucocorticoid receptor deficit [58]. Of interest, we identified familial linkage to and association with T2D and MDD in the glucocorticoid receptor (*GR* or *NR3C1*) gene [59]. However, the above-mentioned findings could also be due to—at least in some patients—variants in the melanocortin receptor 2 (*MC2R*) gene, which might increase responsiveness to ACTH; in fact, we reported *MC2R* linkage and association to/with T2D; one variant was the opposite allele of the variant causing glucocorticoid deficiency syndrome [60].

However, in T2D, even though cortisol is increased, plasma ACTH values are higher, but not significantly compared to controls without T2D. Even considering the wide variability in ACTH, hypercortisolemia without a significant increase in ACTH suggests a peripheral rather than central alteration, such as hyperactivity of the CRH–NE system.

Among the T2D subjects, cortisol levels during the DEX/CRH test are also significantly positively associated with Hba1c, independent of age, body mass index, hypertension, and dyslipidemia [50].

The diurnal rhythm of cortisol shows a peak 30 min after awakening (cortisol awakening response [CAR]) and a decline during the day with a nadir at midnight [61]. Of note, in healthy subjects, increased fasting cortisolemia at 9 am is associated with glycemia at fasting and 2 h status post oral glucose tolerance test, triglyceride levels, and systolic blood pressure [62], and 24 h urinary free cortisol is associated with insulin resistance, visceral obesity, and lipids [63].

Long-lasting cortisol excess of Cushing’s syndrome as well as glucocorticoid treatment cause T2D [64,65]. A flatter diurnal cortisol slope is associated with T2D-related traits (e.g., central adiposity [66], increased cardiovascular disease risk [67]). However, the reported T2D associations with diurnal cortisol patterns are inconsistent. In 3508 adults (50–74 years), inclusive of 238 T2D patients, T2D was associated with a flatter diurnal cortisol slope decline and higher bedtime cortisol, independently from several covariates. However, no association was found between T2D and morning cortisol or CAR [68]. In this study, T2D patients showed a flatter diurnal cortisol slope than healthy subjects, even after adjusting for several potential confounders (e.g., fatigue, BMI, smoking, age, sex, waking time, late saliva collection, employment grade, history of coronary artery disease, cardiovascular medication). A flatter diurnal cortisol slope can be due to low cortisol values on waking or high cortisol values in the evening. T2D patients, compared to control subjects, on average lack significantly higher cortisol waking levels and also have significantly higher bedtime cortisol levels, even after adjusting for covariates [68]. As raised late-night cortisol levels are diagnostic for Cushing’s syndrome [69], to exclude Cushing’s syndrome patients, subjects with very high cortisol were excluded. Obesity, which is strongly associated with Cushing’s syndrome [68], is not driving the results. High late-night salivary cortisol values are reported in T2D without Cushing’s syndrome in other studies as well [70]. Also, feelings of tension and anger are associated with flatter diurnal cortisol rhythms, primarily due to their association with higher evening cortisol [71].

Another study showed that T2D patients, compared to healthy subjects, have smaller hippocampal volumes and show a blunted CAR [72]. A study showed that adolescents with insulin resistance have a blunted CAR, smaller hippocampal volumes, and greater frontal lobe atrophy, as compared to control subjects. A smaller CAR is related to higher BMI, associated with higher fasting insulinemia, which is *per se* associated with smaller hippocampal volume and greater frontal lobe atrophy. Thus, HPA impairment may impact brain structures via metabolic abnormalities [73].

Computed tomography (CT) evaluating the adrenal volume in obese patients with and without T2D reports that total adrenal volume is significantly higher in T2D patients versus control subjects and that visceral fat, visceral fat/subcutaneous fat ratio, and total adrenal volume are highly correlated in all subjects tested. These data suggest that visceral obesity, T2D, and enlarged adrenal glands are associated and support that the hypothesized HPA axis hyperactivation in obese subjects may be involved in T2D pathogenesis [74].

A hereditary predisposition to dysfunction of the HPA axis could therefore induce CRH-noradrenergic system abnormalities, causally contributing to depression, T2D, and depression–T2D comorbidity.

## 3. *CRHR1* Genetic Studies

CRHR1 is an adenylate cyclase-associated membrane receptor and is highly expressed in the neocortex, hippocampus, amygdala, cerebellum, and anterior pituitary gland. *Crhr1* knockout mice show adrenal medullary atrophy, suggesting the peripheral importance of Crhr1 in catecholamine biosynthesis, independent of the central activation of the HPA axis and SNS. Dysfunctional CRHR1 receptors may therefore be responsible for reinforcing the positive feedback of CRH and SNS, peripherally and centrally, with hypersecretion of CRH, bypassing the negative feedback of glucocorticoid receptors. *Crhr1* knockout mice have a depressed HPA axis, low plasma corticosteronemia, and a decreased ACTH and corticosterone response to stress due to marked agenesis of the adrenal glands due to ACTH insufficiency; agenesis is in fact avoided by the administration of ACTH. *CRHR1* variants, therefore, can reduce ACTH release, while catecholamines can positively modulate CRH-dependent ACTH secretion. *Crhr1* knockout mice show, in addition to a reduced stress response, reduced anxious behavior and increased exploratory activity [28,75]. CRHR1 is therefore fundamental in the development of the HPA axis and in the modulation of the anxiety response and behavior, but, in mice, Crhr1 is not essential for the formation of corticotrope cells or for ACTH production in basal conditions [28].

In a study of *Crhr1* knockout mice, Crhr1 appeared to have a bivalent action: its activation in the forebrain glutaminergic system promotes anxious behaviors, whereas in the mesencephalic dopaminergic system, it reduces them. In fact, in mice, while *Crhr1* knockout in prosencephalic glutaminergic neurons leads to reduced anxious behavior and alters neurotransmission in the amygdala and hippocampus, *Crhr1* knockout in mesencephalic dopaminergic neurons leads to the opposite behavior and reduces the release of dopamine by the prefrontal cortex. Thus, CRHR1 may play a bidirectional role in anxiety, as an imbalance between CRHR1-controlled anxiogenic glutamatergic and anxiolytic dopaminergic systems may lead to emotional impairments [76].

Other studies have reported that in young rhesus monkeys, *CRHR1* variants correlate with anxious behavior and brain metabolic activity. Rhesus monkeys’ trait anxiety is similar to the childhood risk trait underlying human anxiety and depression. Single nucleotide polymorphisms (SNPs) of *CRHR1* modulate metabolic activity in the intraparietal sulcus, precuneus, amygdala, and anterior hippocampus, thereby influencing key neural structures of anxious behavior and contributing to psychopathology triggered by childhood trauma [77].

Further work has confirmed that the *CRHR1* gene is associated with depression, increasing the risk of depression after childhood trauma [37,78] and the risk of suicide in males [79]. Three main haplotypes depend on the *CRHR1* SNPs rs7209436, rs4792887, and rs110402 (i.e., CCG, 35.3%, CTG, 32.9%, TCA, 30.4%). The *CRHR1*-rs110402 or TCA haplotype contributes to MDD in child abuse cases [80]. MDD significantly associates with the allelic and genotypic rs242939 SNP and the GGT haplotype formed by the rs1876828, rs242939, and rs242941 alleles [81]. Very anxious subjects suffering from MDD who are homozygous carriers of the GAG haplotype are more sensitive to the antidepressant fluoxetine or desipramine; even highly anxious MDD patients and carriers of the rs242941 G/G genotype associated with the homozygous GAG haplotype have a high response to fluoxetine [82,83].

We previously found no studies on human *CRHR1* and T2D. We were the first to report the role of *CRHR1* in familial T2D, familial MDD, and T2D–MDD [84]. Of note, the *CRHR1* 17q12 locus is associated with T2D [85] and metabolic syndrome [86]. Pancreatic beta cells express CRHR1 on their surface, which stimulates cell proliferation and insulin secretion in a glucose-dependent manner [87]. An alteration in the CRHR1 receptor could therefore be a cause of hyperglycemia and T2D.

## 4. *CRHR2* Genetic Studies

The *CRHR2* gene is expressed in the brain; *Crhr2* knockout mice show reduced stress response [88], hypersensitivity to stress, and anxiety behavior [89,90]. The CRHR2 receptor has urocortins 1, 2, and 3 as ligands [91]. Studies with urocortin analogues have highlighted a strategic role of CRHR2 in glucose homeostasis: it reduces insulin secretion and inhibits gastric emptying and glucose absorption [92] both directly and indirectly through the strengthening of mechano-sensitive gastric vagal afferents [93]; furthermore, the CRHR2 receptor is abundantly expressed at the muscle level and increases insulin sensitivity in this tissue [94,95]. Male *Crhr2* knockout mice develop hepatic steatosis and metabolic syndrome [96]. A few studies have investigated the potential involvement of *CRHR2* in depressive disorders, finding a positive correlation of some alleles with MDD [97] or its mediated resistance to pharmacological treatment [98]. The *CRHR2* 7p21-p15 locus is, however, related to T2D [99,100]; blood glucose, high-density lipoprotein, and triglyceride values [101]; MDD [102]; and bipolar disorder [103,104]. Indeed, bipolar disorder and depression share various genes [105]. *Crhr2* knockout mice have arterial hypertension and reduced sustained hypophagia [88]; thus, *CRHR2* variants might contribute to hypertension and obesity. A recent study showed an association between *CRHR2* SNPs and T2D in a body of pooled genotypic data from 32 genome-wide association studies of European ancestry (GWAS) [106]. We were the first to report the *CRHR2* linkage and association with familial T2D, MDD, and T2D–MDD comorbidity [107].

## 5. Conclusions

HPA axis dysregulation may contribute to T2D and MDD, alone or in combination, due to dysfunctional CRHR1 and CRHR2 receptors. According to our hypothesis, an impaired function of CRHR1 would fuel the CRH–NE–CRH circuit, supporting the hyperactivation of the HPA axis with increased secretion of NE and cortisol, the latter especially implicated in metabolic and depressive disorders. The CRHR2 receptor also appears to be related to metabolic and depressive disorders. Reduced functioning of CRHR2 could contribute to the pathogenesis of T2D and MDD. Future studies to investigate our hypothesis are warranted, including genetic studies of the *CRHR1* and *CRHR2* genes in various ethnicities.
